# A neural network approach to sarcopenia prediction based on bioelectrical impedance in community-dwelling older adults

**DOI:** 10.1371/journal.pone.0335601

**Published:** 2025-11-03

**Authors:** Kyohei Shibuya, Yujiro Asano, Koki Nagata, Taishi Tsuji, Kotaro Kawajiri, Tomohiro Okura

**Affiliations:** 1 Institute of Health and Sport Sciences, University of Tsukuba, Tsukuba, Ibaraki, Japan; 2 Graduate School of Comprehensive Human Sciences, Doctoral Program in Public Health, University of Tsukuba, Tsukuba, Ibaraki, Japan; 3 Doctoral Program in Physical Education, Health and Sport Science, Graduate School of Comprehensive Human Sciences, Degree Programs in Comprehensive Human Sciences, University of Tsukuba, Tsukuba, Ibaraki, Japan; 4 Department of Physical Activity Research, National Institutes of Biomedical Innovation, Health and Nutrition, Settsu, Osaka, Japan; 5 Department of Frailty Research, Center for Gerontology and Social Science, National Center for Geriatrics and Gerontology, Obu, Aichi, Japan; 6 Department of Epidemiology and Prevention, Center for Clinical Sciences, National Center for Global Health and Medicine, Shinjuku-ku, Tokyo, Japan; 7 R&D Center for Tailor-Made QOL, University of Tsukuba, Tsukuba, Ibaraki, Japan; 8 AIZOTH Inc., Tsukuba, Ibaraki, Japan; 9 Department of Complex Systems Science, Graduate School of Informatics, Nagoya University, Nagoya, Aichi, Japan; 10 International Institute for Integrative Sleep Medicine, University of Tsukuba, Tsukuba, Ibaraki, Japan; Niigata University, JAPAN

## Abstract

This study aimed to apply a neural network to raw bioelectrical impedance analysis data and to test whether sarcopenia could be predicted with high accuracy. The study population comprised 727 community-dwelling older adults aged 65–85 years who participated in the Kasama Study from 2015 to 2018. Sarcopenia was determined using the standard values set by the Asian Working Group for Sarcopenia 2019. Skeletal muscle mass index, grip strength, and five-times sit-to-stand test (Dataset 1) or skeletal muscle mass index, grip strength, and gait speed (Dataset 2) were used. The characteristic variables were sex, age, height, and body mass index, as well as parameters from bioelectrical impedance analysis, such as reactance, resistance, and impedance for six frequencies (1, 5, 50, 250, 500, and 1000 kHz) in six body parts measured using a multi-frequency body composition analyzer (MC-980A, Tanita). For analysis, a neural network was used to construct a model. For verification of the model’s accuracy, a receiver operating characteristic analysis was performed to calculate the sensitivity, specificity, area under the curve, and positive and negative predictive values. Among the participants analyzed, 21 (3.3%) in Dataset 1 and 24 (3.7%) in Dataset 2 had sarcopenia. In Dataset 1, the model that used 5, 50, and 250 kHz showed the highest prediction accuracy (sensitivity: 1.00, specificity: 0.91, area under the curve: 0.96, accuracy: 0.91, positive predictive value: 0.28, negative predictive value: 1.00). In Dataset 2, the model that used 50 kHz exhibited the highest prediction accuracy (sensitivity: 0.91, specificity: 0.84, area under the curve: 0.88, accuracy: 0.84, positive predictive value: 0.17, negative predictive value: 1.00). In conclusion, highly accurate predictions are possible by applying a neural network to the raw data obtained from bioelectrical impedance analysis. As a highly accurate sarcopenia screening method, it is expected to be used in various settings, ranging from clinical practice to local communities.

## Introduction

Sarcopenia is defined as age-related loss of muscle mass and function [1,2]. According to the Asian Working Group for Sarcopenia (AWGS) criteria for sarcopenia diagnosis, skeletal muscle mass index (SMI), grip strength, and physical performance can be assessed. When all three measures are below their respective thresholds, the condition is classified as severe sarcopenia [1]. The prevalence of sarcopenia has been reported to be 10% and 18% according to the European Working Group on Sarcopenia in Older People 2 and Asian Working Group for AWGS criteria, respectively [3]. Sarcopenia is associated with falls and fractures [4] and is a risk factor for poor health outcomes, including disability, dementia, and death [5,6]. Given that medical costs for managing patients with sarcopenia may increase [7], the prevention of sarcopenia is an important issue from the perspective of quality of life and social welfare of the older adults. Sarcopenia is associated with poor prognosis, interventions such as resistance training, exercise, and nutritional have been suggested by previous studies to improve early-stage sarcopenia; therefore, early detection and intervention are important issues [8,9].

The diagnosis of sarcopenia is based on skeletal muscle mass measurement using bioelectrical impedance analysis (BIA) or dual-energy X-ray absorptiometry (DXA) in conjunction with an assessment of functional capabilities through performance tests such as grip strength, walking speed, and chair stand tests [1]. Nevertheless, performing all of these measurements requires a substantial financial investment, the expertise of a qualified professional, and a considerable amount of space; thus, it is difficult to carry them out. Straightforward screening instruments such as questionnaires are frequently employed to address these issues [10]. However, sarcopenia is overlooked in many cases, and the use of sarcopenia screening tools such as the SARC-F questionnaire which have low sensitivity is cited as a factor for this [11,12]. Therefore, various screening methods have been proposed such as Ishii test [13] and finger – ring test [14].

BIA measurements are utilized in various situations other than for diagnosing sarcopenia, and body composition can be used to evaluate the state of physical health. To estimate muscle mass and body fat mass, BIA employs measurements such as resistance (R), which represents the resistance to water; reactance (X), which denotes the electrostatic capacity of cell membranes; and impedance (Z), which is the combination of R and X [15]. Phase angle (PhA) is a clinically established BIA parameter that is widely used as an indicator of cellular health. PhA can be calculated as its arc tangent: (X/R) 180/p [16]. Previous studies have linked the PhA, or muscle quality, to nutritional status [16], physical function [17], and adverse health outcomes, including mortality [18]. Furthermore, PhA has been recognized as a potential predictor of sarcopenia [19] and has also been suggested to be a useful indicator for identifying sarcopenia (area under the curve [AUC]: 0.718, 95% confidence interval [CI]: 0.652–0.784 in men and AUC: 0.721, 95% CI: 0.669–0.773 in women) in Japanese older adults, with PhA being reportedly low in patients with sarcopenia [20].

Using multi-frequency BIA, measurements are obtained using a range of frequencies. Low frequencies mainly pass through the resistive extracellular component, whereas high frequencies penetrate cell membranes [21]. The combination of low and high frequencies allows for the differentiation between cellular water distribution and cell membrane conditions [22], thereby increasing the potential for more accurate biological data capture [23]. As suggested by previous reports, observing the raw data (resistance, reactance, and PhA) obtained from multi-frequency BIA is advisable to evaluate physiological changes [24]. However, variables used for sarcopenia prediction have been limited to PhA, select parameters from bioelectrical impedance vector analyses, specific body regions, or certain frequencies; studies that incorporate all these variables are lacking.

In BIA, the number of variables obtained depends on the combination of frequency and measurement site. As the correlation between variables is high, it is challenging to make valid predictions using conventional analysis methods because of issues such as multicollinearity. Consequently, in recent years, an interest in neural networks, a category of machine learning, has increased. Neural networks are algorithms that emulate the human nervous system, enabling the modeling of intricate relationships among numerous variables [25]. A previous study used clinical data to predict sarcopenia in patients undergoing peritoneal dialysis and reported that the accuracy of sarcopenia prediction was higher with models that applied machine learning, such as neural networks and support vector machines, than with models that applied conventional logistic regression analysis [26]. Because the raw data obtained from the BIA are believed to reflect biological information, it is hypothesized that high-precision sarcopenia prediction is possible by modeling these variables using machine learning.

The current study aimed to predict sarcopenia using a maximum of 144 BIA measurements and to develop a more convenient and accurate screening method in order to facilitate early sarcopenia detection in healthy older adults. We propose that developing a screening method based solely on BIA—without the need for additional physical function measurements—will extend the utility of BIA across clinical settings, sports facilities, and municipal programs, thereby enhancing sarcopenia screening efforts.

## Materials and methods

### Study design and participants

This study was conducted with individuals who participated in the Kasama Study from 02/07/2015 to 10/07/2018 and the original data was accessed on 02/12/2022. The Kasama Study is an open cohort study conducted annually since 2009. Individuals were randomly selected from the basic resident register and invited to participate in the study [27]. Those who participated in exercise classes held in the city were invited to participate in this study. Data from the initial participation in health checkups were processed as cross-sectional data, with a total of 724 individuals participating (S1 Fig). The inclusion criteria were as follows: individuals aged ≥65 years who resided in the area and had not received support or nursing care certification. The exclusion criteria were as follows: (1) individuals aged ≤64 years or ≥86 years (n = 16); (2) those with missing BIA data (n = 15); and (3) those with missing physical performance test data (data set 1: n = 38, data set 2: n = 24).

This study was conducted in accordance with the ethical standards of the Helsinki Declaration and was approved by the Ethics Committee of the University of Tsukuba (Tai 30–5). The participants were provided with written and verbal explanations of the study, and only those who provided consent were included.

### Criteria for the diagnosis of sarcopenia

Grip strength was quantified using a grip-strength meter (T.K.K. 5401; Takei Scientific Instruments, Tokyo, Japan). Measurement was performed with the participants in the standing position, with their arms extended along the body. The maximum value of the two measurements on each side was considered the most representative value.

The five-times sit-to-stand test (STS-5) involved standing up from a seated position, standing upright, and then sitting back down in the chair five times. The participants were instructed to repeat these movements as quickly as possible; the test was performed once as a practice run and twice as the actual test. The lowest value was taken as the representative value.

In regard to the 5-meter normal walking test, as a general rule, a straight course of 11 m was marked out, and the time required to traverse the 3–8 m segment was recorded. The participants were instructed to walk at their usual, comfortable speed. Measurements were conducted twice, and the lowest value was regarded as the representative value. All measurements were conducted by well-trained staff. The AWGS 2019 criteria were applied to obtain the skeletal muscle mass index (SMI) from the BIA for the diagnosis of sarcopenia. The standard values were set at <7.0 kg/m^2^ in men and <5.7 kg/m^2^ in women. Grip strength was less than 28 kg for men and less than 18 kg for women. The 5-time chair-stand test was completed in 12 s or more; walking speed was calculated by the formula for a normal walking speed of 1.0 m/s, which was defined as a decrease in walking speed [1]. This was used to determine whether there was a decrease in SMI or grip strength (Dataset 1), and walking speed was used to determine whether there was a decrease in SMI or grip strength (Dataset 2).

### Predictor variables

The basic attributes used as predictor variables were sex, age, height, body mass index (BMI), and BIA parameters. The BIA was conducted using a multi-frequency body composition analyzer (MC-980A, Tanita) [28]. Details on the BIA measurements can be found in our previous study [29]. Although these were not used as predictor variables, information on medical history (heart disease, cerebrovascular disease, kidney disease, and arthralgia)—which may influence BIA measurements—was collected via questionnaire.

The following parameters were measured: body weight, BMI (body weight [kg]/ height [m^2^]), and raw data on reactance, resistance, impedance, and PhA for six frequencies (1, 5, 50, 250, 500, and 1000 kHz) in six body parts (whole body, left and right arms, left and right legs, and lower limbs). As a preliminary data processing step, min–max normalization was performed such that the minimum value of the predictor variable was 0 and the maximum value was 1.

### Data analysis

A total of five models were created using different combinations of frequencies within raw data parameters of the predictor variables—namely, (1) one model with all frequencies from 1 to 1000 kHz; (2) one model with three frequencies of 5, 50, and 250 kHz; (3) one model with a single frequency of 5 kHz; (4) one model with a single frequency of 50 kHz; and (5) one model with a single frequency of 250 kHz. Considering the selection of frequencies, Model (1) employed all available settings—1, 5, 50, 250, 500, and 1000 kHz. Model (2) adopted the 5, 50, and 250 kHz frequencies used in prior studies, since combining low and high frequencies could allow evaluation of intra- and extracellular water distribution [30]. Regarding Models (3)– (5), each used a single frequency to assess predictive performance at that specific frequency.

The basic attributes were input into all models. In the model creation process, the original dataset was divided into two distinct subsets: training data (70%) and validation data (30%). In the training dataset, oversampling was performed to replicate the data of patients with sarcopenia to enhance the prediction accuracy for datasets with an imbalance in the number of observations. A neural network was run using the Multi-Sigma software (AIZOTH Inc., 2023) to create the prediction model. Detailed information on the neural network are presented in previous studies [31,32]. Neural network parameters were manually configured with a single hidden layer comprising 29 neurons, and the output layer’s activation function was set to the sigmoid. Although automatic hyperparameter tuning was attempted, pronounced overfitting led to a drop in test‐set performance, thereby we reverted to manual parameter selection.

**Fig 1:**
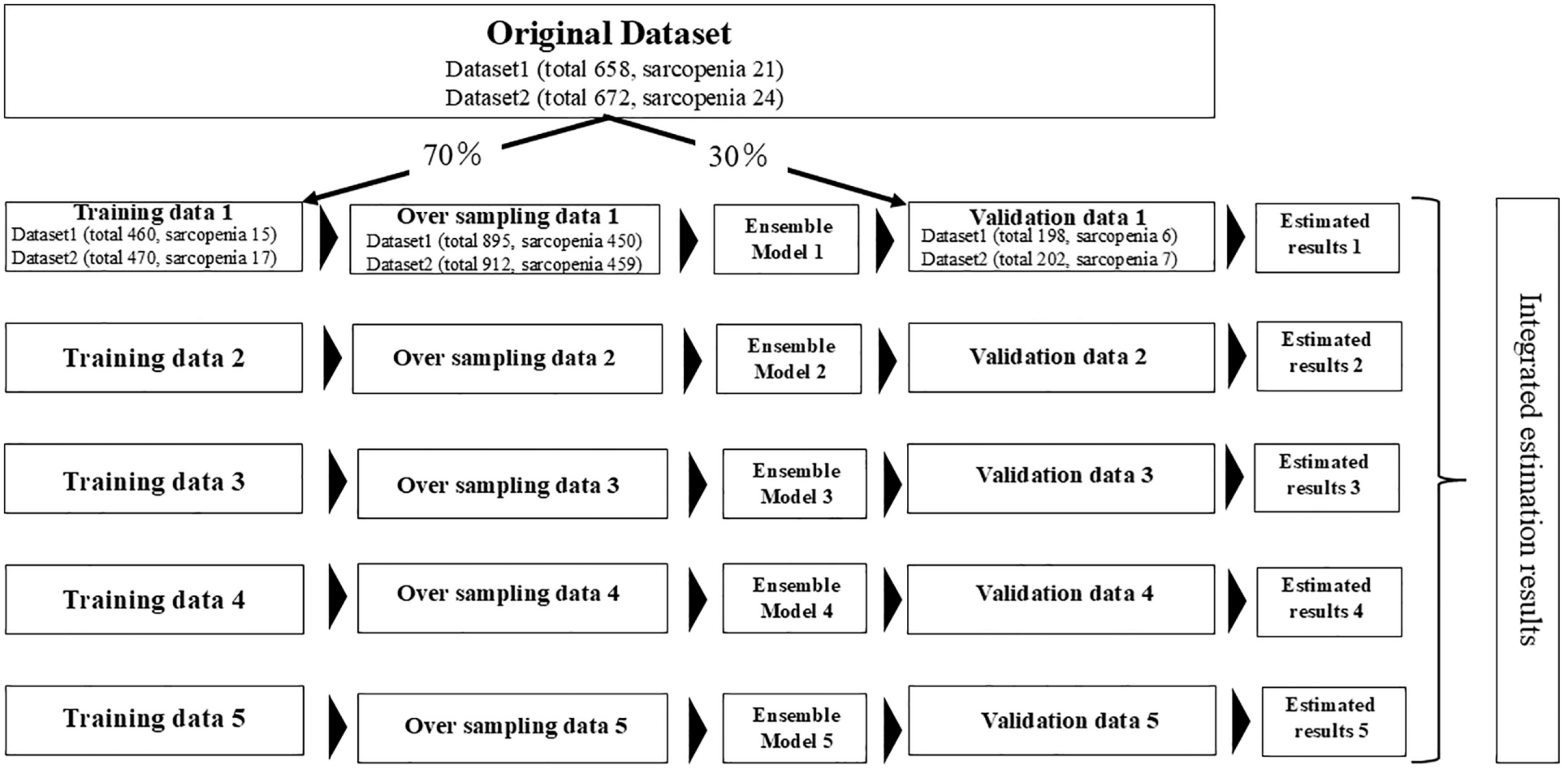
Steps for creating the prediction model.

After creating the prediction model using the training data, its accuracy was calculated using the validation data. These procedures were independently repeated five times. In each iteration, the model was trained on the training set and evaluated on the validation set (Fig 1). The model was evaluated as the mean ± standard deviation across the five repeats using the following metrics: sensitivity, specificity, AUC, accuracy, positive predictive value (PPV), and negative predictive value (NPV). Furthermore, factor analysis based on the partial derivative method [33], which has shown good performance in factor analysis for a neural network model, was conducted to ascertain the extent to which each variable contributed to the prediction value.

For sensitivity analysis, the Wilcoxon rank-sum test was used to determine whether there was a difference in predictive probability between sarcopenia-positive and sarcopenia-negative participants. Because there were relatively many false positives in this study owing to the small number of sarcopenia-positive cases, the variation in the PPV was large. If the physical function and SMI values of participants predicted to be sarcopenia-positive were worse than those of participants predicted to be sarcopenia-negative, the reliability of the prediction model could be confirmed. Therefore, the Wilcoxon rank-sum test was conducted to determine whether there was a difference in performance tests between the positive and negative predictors. Analysis was performed using Python software version 3.11.1.

## Results

A total of 727 individuals participated in this study from 2015 to 2018. We excluded 16 individuals whose age was outside the criteria, 15 individuals with missing BIA data, 38 individuals with missing physical function test data in Dataset 1, and 24 individuals with missing physical function test data in Dataset 2. Ultimately, the final analysis included 658 participants in Dataset 1 and 672 participants in Dataset 2 (S1 Fig).

The characteristics of the participants are summarized in Table 1 and S1 Table. In Dataset 1, the mean age was 73.7 ± 5.2 years, with women accounting for 58.5% (n = 385). In Dataset 2, the mean age was 73.7 ± 5.3 years, with women accounting for 58.6% (n = 394). The percentage of participants with sarcopenia was 3.3% (n = 21) in Dataset 1 and 3.7% (n = 24) in Dataset 2.

**Table 1:** Clinical characteristics of the participants.

		Dataset 1		
		Overall	No sarcopenia	Sarcopenia
	Category	n = 658	n = 637	n = 21
Sex, n (%)	Women	385 (58.5)	381 (59.8)	4 (19.0)
Age (years)		73.7 (5.2)	73.6 (5.2)	78.0 (4.6)
Height (cm)		155.9 (8.4)	155.9 (8.4)	157.2 (6.8)
Weight (kg)		56.6 (9.7)	56.7 (9.8)	51.7 (7.4)
BMI (kg/m^2^)		23.2 (3.1)	23.3 (3.1)	20.9 (2.3)
Resistance at 5 kHz (ohm)		503.6 (67.8)	501.2 (65.8)	577.8 (84.8)
Reactance at 5 kHz (ohm)		16.5 (4.0)	16.5 (4.1)	16.6 (3.3)
Resistance at 50 kHz (ohm)		458.6 (62.6)	456.1 (60.4)	533.6 (82.7)
Reactance at 50 kHz (ohm)		36.8 (7.0)	36.8 (7.0)	37.3 (5.9)
Resistance at 250 kHz (ohm)		420.1 (58.1)	417.6 (55.7)	493.9 (79.9)
Reactance at 250 kHz (ohm)		32.3 (5.2)	32.2 (5.2)	34.5 (4.9)
Impedance at 5 kHz (ohm)		503.9 (67.8)	501.4 (65.8)	578.1 (84.9)
Impedance at 50 kHz (ohm)		460.1 (62.7)	457.6 (60.5)	534.9 (82.6)
Impedance at 250 kHz (ohm)		421.3 (58.3)	418.9 (55.9)	495.1 (80.0)
PhA at 5 kHz (°)		1.9 (0.4)	1.9 (0.4)	1.7 (0.3)
PhA at 50 kHz (°)		4.6 (0.7)	4.6 (0.7)	4.0 (0.6)
PhA at 250 kHz (°)		4.4 (0.6)	4.4 (0.6)	4.0 (0.4)
Heart disease, n (%)		70 (10.6)	69 (10.8)	1 (4.8)
Cerebrovascular disease, n (%)		24 (3.6)	22 (3.5)	2 (9.5)
Kidney disease, n (%)		22 (3.3)	21 (3.3)	1 (4.8)
Arthralgia, n (%)		221 (33.6)	211 (33.1)	10 (47.6)
Decreased SMI, n (%)	Men: < 7.0 kg/m^2^ Women: < 5.7 kg/m^2^	101 (15.3)	80 (12.6)	21 (100.0)
Decreased grip strength, n (%)	Men: < 28 kg Women: < 18 kg	50 (7.6)	32 (5.0)	18 (85.7)
Decreased STS-5, n (%)	≥12 sec	26 (4.0)	18 (2.8)	8 (38.1)

BMI, body mass index; SMI, skeletal muscle mass index; PhA, phase angle.

Resistance, reactance, impedance, and PhA indicate values for the entire body. Values are presented as mean (standard deviation), unless otherwise indicated.

**Fig 2:**
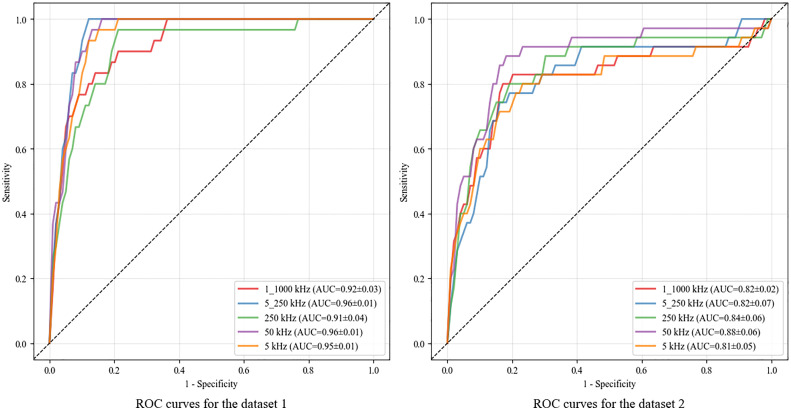
Receiver operating characteristic curve for each model. AUC: Area under the curve indicates the ensemble average ± standard deviation over five resampling iterations.

The performance values for sarcopenia prediction by the five models integrated in the ensemble average of their prediction values are presented in Table 2. All models showed good predictive performance (AUC > 0.80) (Fig 2). In Dataset 1, the model that used 5, 50, and 250 kHz exhibited the highest prediction accuracy (sensitivity: 1.00 ± 0.00, specificity: 0.91 ± 0.03, AUC: 0.96 ± 0.01, accuracy: 0.91 ± 0.03, PPV: 0.28 ± 0.10, and NPV: 1.00 ± 0.00). In Dataset 2, the model that used 50 kHz demonstrated the highest prediction accuracy (sensitivity: 0.91 ± 0.13, specificity: 0.84 ± 0.04, AUC: 0.88 ± 0.06, accuracy: 0.84 ± 0.04, PPV: 0.17 ± 0.03, and NPV: 1.00 ± 0.01).

**Table 2:** Model prediction accuracy.

	Sensitivity	Specificity	AUC	Accuracy	PPV	NPV
	Mean ± standard deviation (minimum and maximum)					
Dataset 1						
1–1000 kHz	0.93 ± 0.09 (0.83, 1.00)	0.83 ± 0.11 (0.66, 0.95)	0.92 ± 0.03 (0.89, 0.97)	0.83 ± 0.11 (0.67, 0.95)	0.19 ± 0.11 (0.08, 0.36)	1.00 ± 0.00 (0.99, 1.00)
5, 50, and 250 kHz	1.00 ± 0.00 (1.00, 1.00)	0.91 ± 0.03 (0.88, 0.96)	0.96 ± 0.01 (0.95, 0.98)	0.91 ± 0.03 (0.88, 0.96)	0.28 ± 0.10 (0.21, 0.46)	1.00 ± 0.00 (1.00, 1.00)
250 kHz	0.97 ± 0.07 (0.83, 1.00)	0.85 ± 0.04 (0.80, 0.90)	0.91 ± 0.04 (0.84, 0.96)	0.85 ± 0.04 (0.8, 0.9)	0.17 ± 0.03 (0.13, 0.21)	1.00 ± 0.00 (0.99, 1.00)
50 kHz	1.00 ± 0.00 (1.00, 1.00)	0.90 ± 0.04 (0.84, 0.95)	0.96 ± 0.01 (0.95, 0.97)	0.90 ± 0.04 (0.84, 0.95)	0.25 ± 0.08 (0.16, 0.38)	1.00 ± 0.00 (1.00, 1.00)
5 kHz	1.00 ± 0.00 (1.00, 1.00)	0.86 ± 0.04 (0.80, 0.89)	0.95 ± 0.01 (0.93, 0.97)	0.86 ± 0.04 (0.8, 0.89)	0.19 ± 0.04 (0.13, 0.22)	1.00 ± 0.00 (1.00, 1.00)
Dataset 2						
1–1000 kHz	0.83 ± 0.06 (0.71, 0.86)	0.86 ± 0.05 (0.81, 0.94)	0.82 ± 0.03 (0.78, 0.85)	0.86 ± 0.05 (0.81, 0.93)	0.19 ± 0.06 (0.14, 0.29)	0.99 ± 0.00 (0.99, 0.99)
5,50, and 250 kHz	0.89 ± 0.12 (0.71, 1.00)	0.78 ± 0.15 (0.59, 0.96)	0.82 ± 0.08 (0.69, 0.91)	0.79 ± 0.14 (0.60, 0.95)	0.18 ± 0.12 (0.07, 0.38)	1.00 ± 0.00 (0.99, 1.00)
250 kHz	0.83 ± 0.12 (0.71, 1.00)	0.85 ± 0.08 (0.73, 0.93)	0.84 ± 0.07 (0.75, 0.91)	0.85 ± 0.07 (0.74, 0.92)	0.18 ± 0.06 (0.12, 0.26)	0.99 ± 0.00 (0.99, 1.00)
50 kHz	0.91 ± 0.13 (0.71, 1.00)	0.84 ± 0.04 (0.77, 0.87)	0.88 ± 0.06 (0.78, 0.94)	0.84 ± 0.04 (0.78, 0.87)	0.17 ± 0.03 (0.14, 0.21)	1 ± 0.01 (0.99, 1.00)
5 kHz	0.83 ± 0.06 (0.71, 0.86)	0.82 ± 0.09 (0.71, 0.93)	0.81 ± 0.06 (0.75, 0.91)	0.82 ± 0.09 (0.71, 0.93)	0.17 ± 0.08 (0.10, 0.3)	0.99 ± 0.00 (0.99, 0.99)

The mean ± standard deviation (minimum and maximum) values for the ensemble average are shown.

AUC, area under the curve; PPV, positive predictive value; NPV, negative predictive value.

The results of the sensitivity analysis indicated that the predictive probability of sarcopenia was significantly higher in all models for those with sarcopenia than for those without sarcopenia (p < 0.05) (S2 Fig and S3 Fig). A comparison of the performance tests between sarcopenia-positive and sarcopenia-negative predictors is presented in S2 Table. In Dataset 1, significant differences in all items were found for the model using 1–1000 kHz; however, there were no significant differences in the SMI (using250kHz: p = 0.089, 50kHz: p = 0.091, 5kHz: p = 0.474) or grip strength (using 5, 50 and 250kHz: p = 0.195, 250kHz: p = 0.270, 50kHz: p = 0.259, 5kHz: p = 0.282) and sts-5 (using 50kHz: p = 0.142) portion of the model. In Dataset 2, SMI, grip strength, and gait speed were significantly lower (p < 0.05) in the model using 1–1000 kHz between sarcopenia-positive and sarcopenia-negative predictors. In other models, those predicted to have sarcopenia showed significantly lower values for SMI and gait speed; however, no significant difference in grip strength using 5kHz was found (p = 0.113).

## Discussion

In the current study, we evaluated a simple and highly accurate sarcopenia screening method for identifying sarcopenia in older adults by applying a neural network to analyze raw data from the BIA. Our results indicated that a high degree of accuracy for predicting sarcopenia was achieved using a straightforward measurement approach (AUC = 0.96). The average age of the study participants was 73.7 ± 5.2 years, and the number of participants with sarcopenia according to the AWGS 2019 criteria was 21 (3.3%) in Dataset 1 and 24 (3.7%) in Dataset 2. In a previous study of a similar population (average age: 74.1 years), the prevalence of sarcopenia according to the AWGS 2019 criteria was 6.6% [34]. The prediction accuracy was also confirmed to be sufficient, even in populations with few sarcopenia cases. Its portability, minimal space requirements, and lack of need for specialized training—allowing a single operator to perform the measurement—make it a promising screening tool. Moreover, since BIA is employed not only for sarcopenia screening but also for routine health management, it represents a highly versatile approach.

To date, the most prevalent screening method has been the use of questionnaires such as the SARC-F [12]. While this method is simple, it has low sensitivity [35]. Owing to the limitations of screening questionnaires, various screening methods that utilize objective indicators have been developed, such as the method that uses the calf circumference (AUC: 0.817, 95% CI: 0.74–0.89) [36] and the method that uses age, grip strength, and calf circumference (AUC: 0.94, 95% CI: 0.92–0.96 for men and AUC: 0.91, 95% CI: 0.89–0.93 for women) [13]. These methods show good accuracy but are limited by the need for a specialist measurer. The results of this study not only suggest the possibility of developing space-saving screening methods but also indicate the simplicity of being able to perform measurements by oneself just by getting on the BIA device with high accuracy. Moreover, outperforming the sarcopenia prediction accuracy using PhA was confirmed—a primary BIA parameter (AUC: 0.718, 95% CI: 0.652–0.784 in men and AUC: 0.721, 95% CI: 0.669–0.773 in women) [20]. It was suggested that leveraging multiple BIA parameters could further enhance accuracy. However, the PPV of the created model in this study was low compared to that in previous studies, which is thought to be attributable to the low number of sarcopenia cases among the participants of this study and several false-positive judgments. Therefore, as a sensitivity analysis, we compared the performance tests between positive and negative predictors. The positive predictors performed significantly worse than the negative predictors in the performance test, suggesting that the model is useful for screening high-risk individuals. Since even false-positive cases tended to exhibit poorer SMI and worse performance on physical function tests, a high false-positive rate may nevertheless strain healthcare resources; therefore, further improvements in specificity and overall accuracy are warranted.

In this study, we evaluated different models using the sit-to-stand test and walking speed as indicators of physical performance. In Dataset 1, in which the sit-to-stand test served as the performance indicator, the highest accuracy was achieved when using 5, 50, and 250 kHz (AUC = 0.96). In contrast, in Dataset 2, which used walking speed as the indicator, the highest AUC was obtained with 50 kHz alone (AUC = 0.88). One possible explanation for this discrepancy is the nature of the performance tests themselves. Both the sit-to-stand test and walking speed are known to depend on multiple determinants—such as muscle strength, sensory function, balance, and psychological factors [37,38]. However, since the sit-to-stand test yielded a higher AUC, this suggests that the capabilities assessed by BIA may differ between these two performance measures. Moreover, in both models, excellent predictive accuracy was observed using 50 kHz as a single frequency. Since 50 kHz is a widely used frequency band [39], it is expected to be readily implementable in real‐world settings. In research applications and fine-grained evaluations, multi-frequency methods offer benefits; therefore, frequencies should be selected according to the measurement context.

Low frequencies strongly reflect the state of the extracellular compartment, whereas high frequencies penetrate the myocyte membrane and reflect the state of both the intracellular and extracellular compartments. By applying these principles, the appendicular extracellular-to-intracellular water ratio can be possibly estimated [40]. The appendicular extracellular-to-intracellular water ratio has been reported to be associated with mortality and has gained attention as an indicator of muscle quality [41]. It is thought that by utilizing multiple frequencies in this way, more detailed biological information can be potentially obtained; nevertheless, further research on the relationship between multiple frequency variables obtained using a multi-frequency body composition analyzer and sarcopenia is required. Another advantage is that it is possible to evaluate asymmetry in the body by measuring multiple body parts. Muscle strength asymmetry is reportedly a predictor of functional disability [42] and sarcopenia [43]. The use of machine learning for various variables in predicting sarcopenia has resulted in highly accurate outcome predictions [44–46]. In the data used in this study, the correlation coefficients between variables were high; additionally, the use of machine learning, which can consider nonlinear effects, resulted in high prediction accuracy.

The principal strength of this study is the application of machine learning to raw BIA data, specifically analyzed for community-dwelling older adults. Nevertheless, this study had some limitations. First, the participants of this study were individuals who participated in health checkups; thus, it can be assumed that they represent a healthy group. In a previous study including Japanese older adults, 33.9% of men and 48.3% of women were classified as having low SMI, and 24.0% of men and 23.8% of women had low grip strength—rates that are higher than those observed in our current study [47]. It is possible that the results are an underestimation because of the limited number of individuals with sarcopenia. In the future, it would be beneficial to examine different groups. Second, this study used the BIA to assess muscle mass. DXA is considered the gold standard for measuring muscle mass. However, the accuracy of BIA measurements is possibly inferior to that of the gold standard such as DXA. Therefore, this may have led to misclassification of sarcopenia, affecting accuracy, further research is necessary to substantiate these findings. However the muscle mass prediction formula used in this study has been confirmed to have a very high correlation with DXA measurement results (R2 = 0.87 in man and R2 = 0.86 in women) [30]. Third, the results of the factor analysis indicated that Sex, low-frequency reactance and PhA significantly contributed to the overall results (S4 Fig and S5 Fig). The association between reactance across different measurement frequencies and sarcopenia has not been firmly established. Our results may contribute additional insight. However, these results should be interpreted with caution. The correlation coefficients for the variables used in this study were markedly high, suggesting the potential for unreliable results. Further studies are required to facilitate the interpretation of the model. Fourth, water and food intake was not controlled during BIA measurement. Since it has been reported that BIA values change depending on water intake immediately prior to measurement, consideration of these effects will be necessary in future studies. Fifth, accuracy between different BIA has not been confirmed. Since we built our models using raw BIA data across multiple frequencies, we suggest that our approach could be applied to different BIA devices. However, further evaluation is required to determine its accuracy with lower-cost instruments or those from other manufacturers.

## Conclusion

The application of machine learning to raw data obtained from the BIA indicated the potential for highly accurate predictions. BIA is already applied in health checkups, personal health management, and clinical practice and could be utilized as a community-based screening method; nonetheless, further gains in measurement accuracy are required.

## Supporting information

S1 Fig Flowchart of the participants. (DOCX)

S2 Fig Comparison of the predictive probability of sarcopenia-positive and sarcopenia-negative individuals in Dataset 1. (DOCX)

S3 Fig Comparison of the predictive probability of sarcopenia-positive and sarcopenia-negative individuals in Dataset 2. (DOCX)

S4 Fig Factor analysis in Dataset 1. (DOCX)

S5 Fig Factor analysis in Dataset 2. (DOCX)

S1 Table Clinical characteristics of the participants. (DOCX)

S2 Table Comparison of skeletal muscle mass index and performance tests between positive and negative predictors. (DOCX)
