# Supplementary material for: A neural network approach to sarcopenia prediction based on bioelectrical impedance in community-dwelling older adults
Source: PLoS One. 2025 Nov 3;20(11):e0335601. doi: 10.1371/journal.pone.0335601 (PMC12582432; doi:10.1371/journal.pone.0335601)
Supplement: S1 Fig — (DOCX) [file pone.0335601.s001.docx]

**S1 Fig. Flowchart of the participants.**


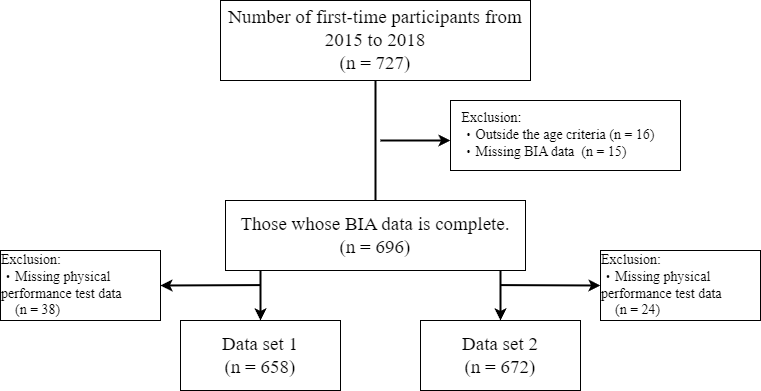


Dataset 1: SMI, grip strength, and 5-repetition chair-stand test were used to determine sarcopenia.

Dataset 2: SMI, grip strength, and walking speed were used to determine sarcopenia.
