# Supplementary material for: A neural network approach to sarcopenia prediction based on bioelectrical impedance in community-dwelling older adults
Source: PLoS One. 2025 Nov 3;20(11):e0335601. doi: 10.1371/journal.pone.0335601 (PMC12582432; doi:10.1371/journal.pone.0335601)

**S****2 Fig. Comparison of the predictive probability of sarcopenia-positive and sarcopenia-negative individuals in Dataset 1.**


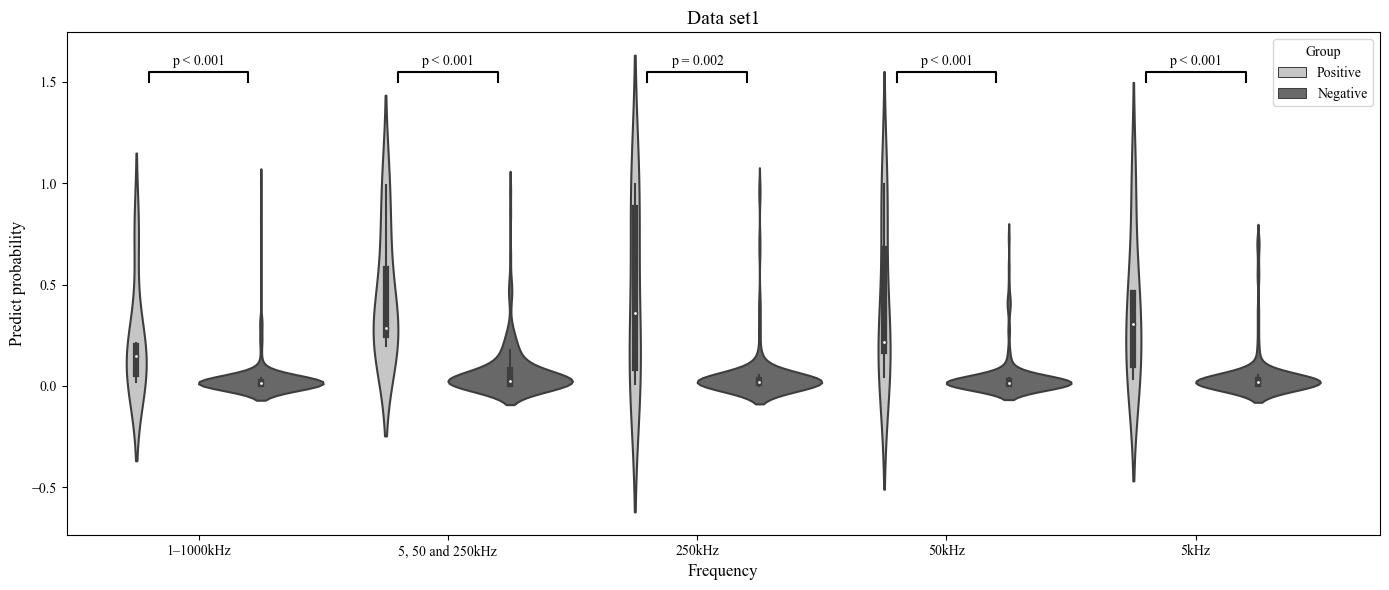

Supplement: S2 Fig — (DOCX) [file pone.0335601.s002.docx]
