# Supplementary material for: A neural network approach to sarcopenia prediction based on bioelectrical impedance in community-dwelling older adults
Source: PLoS One. 2025 Nov 3;20(11):e0335601. doi: 10.1371/journal.pone.0335601 (PMC12582432; doi:10.1371/journal.pone.0335601)
Supplement: S3 Fig — (DOCX) [file pone.0335601.s003.docx]

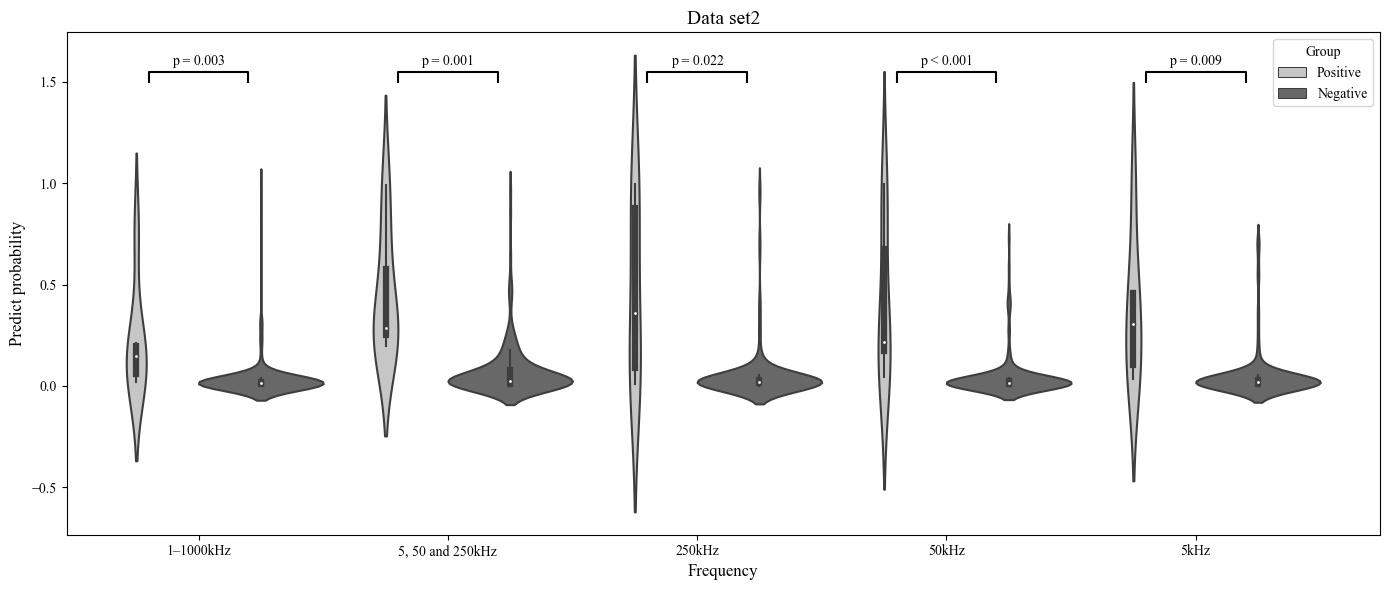
**S3 Fig. Comparison of the predictive probability of sarcopenia-positive and sarcopenia-negative individuals in Dataset 2.**
