# Supplementary material for: A neural network approach to sarcopenia prediction based on bioelectrical impedance in community-dwelling older adults
Source: PLoS One. 2025 Nov 3;20(11):e0335601. doi: 10.1371/journal.pone.0335601 (PMC12582432; doi:10.1371/journal.pone.0335601)
Supplement: S4 Fig — (DOCX) [file pone.0335601.s004.docx]

**S4 Fig.** **Factor analysis in Dataset 1.**


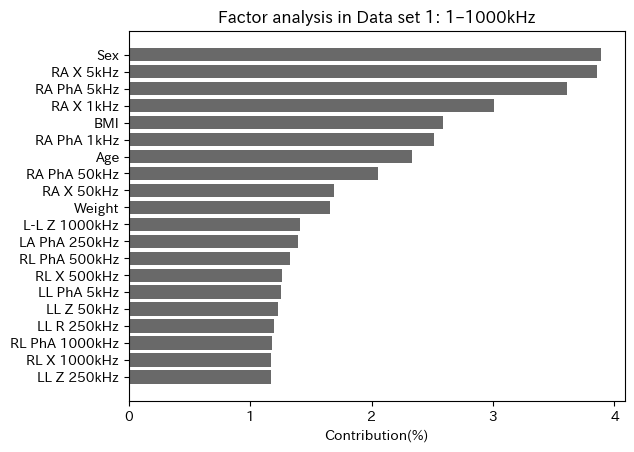

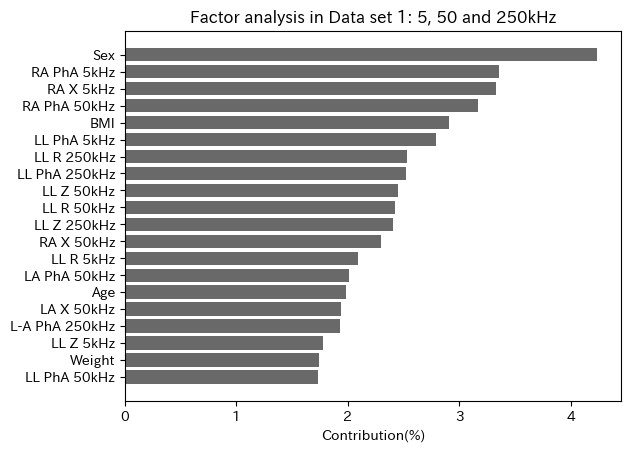

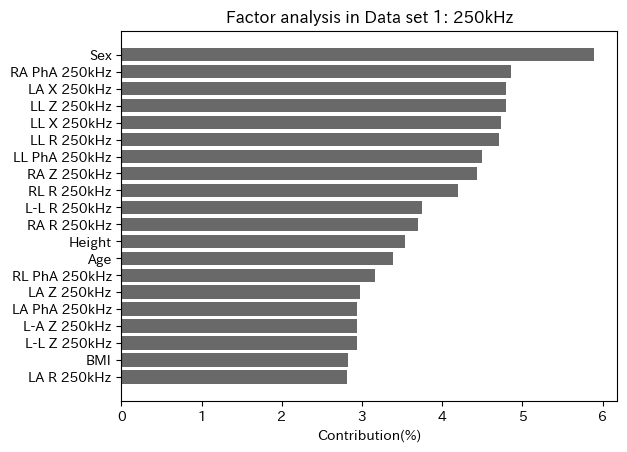

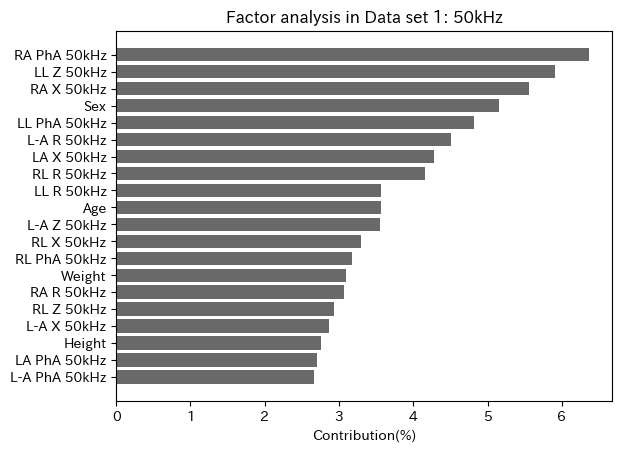

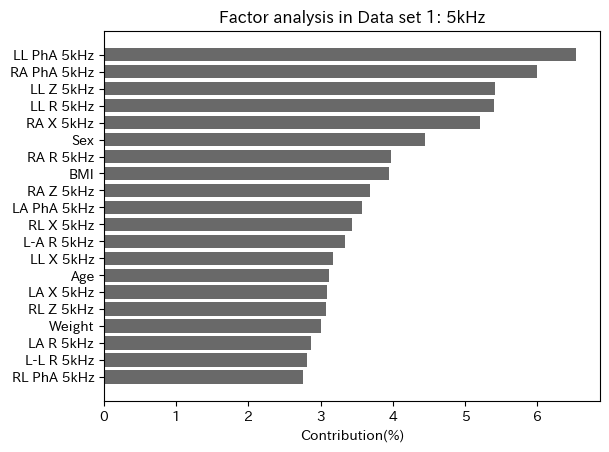


RL, right leg; LL, left leg; RA, right arm; LA, left arm; L-A, whole body; L-L, lower limbs; R, resistance; X, reactance; X, impedance; PhA, phase angle.
