# Supplementary material for: A neural network approach to sarcopenia prediction based on bioelectrical impedance in community-dwelling older adults
Source: PLoS One. 2025 Nov 3;20(11):e0335601. doi: 10.1371/journal.pone.0335601 (PMC12582432; doi:10.1371/journal.pone.0335601)
Supplement: S1 Table — (DOCX) [file pone.0335601.s006.docx]

**S1 Table. Clinical characteristics of the participants.**

|  |  | **Dataset 2** | | |
| --- | --- | --- | --- | --- |
|  |  | **Overall** | **No sarcopenia** | **Sarcopenia** |
|  | **Category** | **n=672** | **n=648** | **n=24** |
| Sex, n (%) | Women | 394 (58.6) | 387 (59.7) | 7 (29.2) |
| Age (years) |  | 73.7 (5.3) | 73.6 (5.2) | 78.4 (4.1) |
| Height (cm) |  | 155.9 (8.3) | 155.9 (8.4) | 155.8 (7.4) |
| Weight (kg) |  | 56.6 (9.8) | 56.9 (9.8) | 50.1 (7.2) |
| BMI (kg/m^2^) |  | 23.2 (3.1) | 23.3 (3.1) | 20.6 (2.2) |
| Resistance 5 kHz (ohm) |  | 502.9 (68.1) | 500.4 (66.2) | 570.1 (86.6) |
| Reactance 5 kHz (ohm) |  | 16.5 (4.1) | 16.5 (4.1) | 15.7 (3.6) |
| Resistance 50 kHz (ohm) |  | 458.1 (62.8) | 455.4 (60.5) | 528.7 (82.5) |
| Reactance 50 kHz (ohm) |  | 36.7 (7.1) | 36.8 (7.1) | 35.2 (6.7) |
| Resistance 250 kHz (ohm) |  | 419.7 (58.3) | 417.1 (55.7) | 490.9 (78.9) |
| Reactance 250 kHz (ohm) |  | 32.2 (5.3) | 32.2 (5.3) | 33.5 (5.2) |
| Impedance 5 kHz (ohm) |  | 503.2 (68.2) | 500.7 (66.2) | 570.3 (86.7) |
| Impedance 50 kHz (ohm) |  | 459.6 (63.0) | 457.0 (60.7) | 529.9 (82.5) |
| Impedance 250 kHz (ohm) |  | 420.9 (58.4) | 418.3 (55.9) | 492.1 (79.0) |
| PhA 5 kHz (°) |  | 1.9 (0.4) | 1.9 (0.4) | 1.6 (0.3) |
| PhA 50 kHz (°) |  | 4.6 (0.7) | 4.6 (0.7) | 3.8 (0.7) |
| PhA 250 kHz (°) |  | 4.4 (0.6) | 4.4 (0.6) | 3.9 (0.4) |
| Heart disease, n (%) |  | 73 (10.9) | 71 (11.0) | 2 (8.3) |
| Cerebrovascular disease, n (%) |  | 25 (3.7) | 23 (3.5) | 2 (8.3) |
| Kidney disease, n (%) |  | 23 (3.4) | 22 (3.4) | 1 (4.2) |
| Arthralgia, n (%) |  | 229 (34.1) | 216 (33.3) | 13 (54.2) |
| Decreased SMI, n (%) | Men: <7.0 kg/m^2^ Women: <5.7 kg/m^2^ | 103 (15.3) | 79 (12.2) | 24 (100.0) |
| Decreased grip strength, n (%) | Men: <28 kg Women: <18 kg | 52 (7.7) | 33 (5.1) | 19 (79.2) |
| Decreased gait speed, n (%) | <1.0 m/s | 27 (4.0) | 17 (2.6) | 10 (41.7) |

BMI, body mass index; SMI, skeletal muscle mass index; PhA, phase angle.

Resistance, reactance, impedance, and PhA indicate values for the entire body. Continuous values are presented as mean (standard deviation).
