# Supplementary material for: A neural network approach to sarcopenia prediction based on bioelectrical impedance in community-dwelling older adults
Source: PLoS One. 2025 Nov 3;20(11):e0335601. doi: 10.1371/journal.pone.0335601 (PMC12582432; doi:10.1371/journal.pone.0335601)
Supplement: S2 Table — (DOCX) [file pone.0335601.s007.docx]

**S2 Table. Comparison of skeletal muscle mass index and performance tests between positive and negative predictors**

|  |  | SMI | | | | | | | |  |  |  | Handgrip strength | | | | | | |  |  |
| --- | --- | --- | --- | --- | --- | --- | --- | --- | --- | --- | --- | --- | --- | --- | --- | --- | --- | --- | --- | --- | --- |
|  | n | Positive  prediction | | |  | n | Negative  prediction | | | p-value | |  | Positive  prediction | | |  | Negative  prediction | | | p-value | |
| Data set1 |  |  |  |  |  |  |  |  |  |  |  |  |  |  |  |  |  |  |  |  |  |
| 1­–1000kHz | 47 | 6.38 | ± | 0.78 |  | 151 | 7.18 | ± | 1.07 | <0.001 | |  | 24.30 | ± | 6.12 |  | 28.11 | ± | 7.27 | 0.001 | |
| 5, 50 and 250kHz | 30 | 6.50 | ± | 0.89 |  | 168 | 7.08 | ± | 1.07 | 0.017 | |  | 25.62 | ± | 7.21 |  | 27.49 | ± | 7.17 | 0.195 | |
| 250kHz | 16 | 6.48 | ± | 0.85 |  | 182 | 7.04 | ± | 1.07 | 0.089 | |  | 24.94 | ± | 7.63 |  | 27.40 | ± | 7.13 | 0.270 | |
| 50kHz | 22 | 6.56 | ± | 0.73 |  | 176 | 7.05 | ± | 1.09 | 0.091 | |  | 25.51 | ± | 7.38 |  | 27.42 | ± | 7.15 | 0.259 | |
| 5kHz | 13 | 6.66 | ± | 0.76 |  | 185 | 7.01 | ± | 1.08 | 0.474 | |  | 24.57 | ± | 5.41 |  | 27.39 | ± | 7.27 | 0.282 | |
| Data set2 |  |  |  |  |  |  |  |  |  |  |  |  |  |  |  |  |  |  |  |  |  |
| 1­–1000kHz | 20 | 6.10 | ± | 0.81 |  | 182 | 7.09 | ± | 1.09 | <0.001 | |  | 23.12 | ± | 6.75 |  | 27.10 | ± | 7.08 | 0.012 | |
| 5,50 and 250kHz | 31 | 6.28 | ± | 0.91 |  | 171 | 7.12 | ± | 1.09 | <0.001 | |  | 23.37 | ± | 6.41 |  | 27.31 | ± | 7.10 | 0.005 | |
| 250kHz | 19 | 6.05 | ± | 0.56 |  | 183 | 7.09 | ± | 1.10 | <0.001 | |  | 22.41 | ± | 7.06 |  | 27.15 | ± | 7.01 | 0.003 | |
| 50kHz | 19 | 6.21 | ± | 0.63 |  | 183 | 7.08 | ± | 1.11 | <0.001 | |  | 23.81 | ± | 7.85 |  | 27.01 | ± | 7.01 | 0.045 | |
| 5kHz | 24 | 6.02 | ± | 0.62 |  | 178 | 7.13 | ± | 1.09 | <0.001 | |  | 22.75 | ± | 6.41 |  | 27.24 | ± | 7.07 | 0.002 | |

**S2 Table. Comparison of skeletal muscle mass index and performance tests between positive and negative predictors (continued)**

|  |  | STS-5 | | | | | | |  |  |  | Gait speed | | | | | | |  |
| --- | --- | --- | --- | --- | --- | --- | --- | --- | --- | --- | --- | --- | --- | --- | --- | --- | --- | --- | --- |
|  | n | Positive  prediction | | |  | Negative  prediction | | | p-value | |  | Positive  prediction | | | |  | Negative  prediction | | p-value |
| Data set1 |  |  |  |  |  |  |  |  |  |  |  |  |  |  |  |  |  |  |  |
| 1­–1000kHz | 47 | 8.48 | ± | 2.11 |  | 7.39 | ± | 1.62 | 0.002 | |  |  |  |  |  |  |  |  |  |
| 5, 50 and 250kHz | 30 | 8.69 | ± | 2.27 |  | 7.46 | ± | 1.65 | 0.005 | |  |  |  |  |  |  |  |  |  |
| 250kHz | 16 | 9.30 | ± | 2.32 |  | 7.51 | ± | 1.68 | 0.002 | |  |  |  |  |  |  |  |  |  |
| 50kHz | 22 | 8.42 | ± | 2.42 |  | 7.55 | ± | 1.69 | 0.142 | |  |  |  |  |  |  |  |  |  |
| 5kHz | 13 | 9.10 | ± | 2.34 |  | 7.55 | ± | 1.72 | 0.017 | |  |  |  |  |  |  |  |  |  |
| Data set2 |  |  |  |  |  |  |  |  |  |  |  |  |  |  |  |  |  |  |  |
| 1­–1000kHz | 20 |  |  |  |  |  |  |  |  |  |  | 1.28 | ± | 0.26 |  | 1.44 | ± | 0.22 | 0.009 |
| 5, 50 and 250kHz | 31 |  |  |  |  |  |  |  |  |  |  | 1.31 | ± | 0.24 |  | 1.44 | ± | 0.22 | 0.002 |
| 250kHz | 19 |  |  |  |  |  |  |  |  |  |  | 1.30 | ± | 0.26 |  | 1.43 | ± | 0.22 | 0.032 |
| 50kHz | 19 |  |  |  |  |  |  |  |  |  |  | 1.27 | ± | 0.26 |  | 1.44 | ± | 0.22 | 0.005 |
| 5kHz | 24 |  |  |  |  |  |  |  |  |  |  | 1.33 | ± | 0.29 |  | 1.43 | ± | 0.21 | 0.113 |

SMI, skeletal muscle mass index; STS-5, The five-times sit-to-stand test
